# Using First Passage Statistics to Extract Environmentally Dependent Amino Acid Correlations

**DOI:** 10.1371/journal.pone.0101665

**Published:** 2014-07-07

**Authors:** Benjamin D. Greenbaum, Pradeep Kumar, Albert Libchaber

**Affiliations:** 1 Departments of Medicine, Division of Hematology and Medical Oncology, and Pathology, and the Tisch Cancer Institute, Icahn School of Medicine at Mount Sinai, New York, New York, United States of America; 2 The Simons Center for Systems Biology, Institute for Advanced Study, Princeton, New Jersey, United States of America; 3 Center for Studies in Physics and Biology, The Rockefeller University, New York, New York, United States of America; 4 Department of Physics, University of Arkansas at Little Rock, Little Rock, Arkansas, United States of America; Rosalind Franklin University, United States of America

## Abstract

In this work, we study the first passage statistics of amino acid primary sequences, that is the probability of observing an amino acid for the first time at a certain number of residues away from a fixed amino acid. By using this rich mathematical framework, we are able to capture the background distribution for an organism, and infer lengths at which the first passage has a probability that differs from what is expected. While many features of an organism's genome are due to natural selection, others are related to amino acid chemistry and the environment in which an organism lives, constraining the randomness of genomes upon which selection can further act. We therefore use this approach to infer amino acid correlations, and then study how these correlations vary across a wide range of organisms under a wide range of optimal growth temperatures. We find a nearly universal exponential background distribution, consistent with the idea that most amino acids are globally uncorrelated from other amino acids in genomes. When we are able to extract significant correlations, these correlations are reliably dependent on optimal growth temperature, across phylogenetic boundaries. Some of the correlations we extract, such as the enhanced probability of finding, for the first time, a cysteine three residues away from a cysteine or glutamic acid two residues away from an arginine, likely relate to thermal stability. However, other correlations, likely appearing on alpha helical surfaces, have a less clear physiochemical interpretation and may relate to thermal stability or unusual metabolic properties of organisms that live in a high temperature environment.

## Introduction

First passage statistics provide a natural mathematical framework for analyzing the likelihood of the first occurrence of an event after some initial event [1]. While many analytical results have been derived using these distributions in several fields, here we present a novel application of these distributions to a problem in genome distributions. Namely, what is the best method, both in terms of the underlying mathematics and empirical application to sequence datasets, to infer the set of amino acid correlations in proteins that are dependent on the environment in which they function.

The genome sequencing of many extremophiles has created an opportunity to probe basic, yet practical questions about how an organism's physiochemical environment affects its genome [2], [3]. Extremophiles have been sequenced across vast evolutionary distances, representing a broad range of environmental conditions. Hence these organisms provide a deep-field lens for resolving how variations in physiochemical environment alter genome characteristics. Motivated by this, several authors have noted the effects of optimal growth temperature (OGT) on various amino acid features [4]–[9].

Globally, the frequencies of most amino acids are unrelated to the context of the amino acid words in which they appear [10]. Moreover, one can show that the observed OGT dependence of amino acid words can often be explained in terms of the OGT dependence of individual amino acids or small subwords [11]. Consequently, we argue that first passage distributions are a natural mathematical language for inferring environmentally dependent correlations between discontiguous residues. In the genomic context, these distributions are the probability of finding an amino acid for the first time at a specific number of residues away from a given amino acid.

We observe a universal exponential background distribution for amino acid first passage distributions across a set of 76 organisms, chosen to represent the range of well-characterized optimal growth temperatures among organisms with fully sequenced proteomes. The exponential background distribution implies that typically there is little genome wide correlation between amino acids. We use this observation to infer an empirical exponential background distribution - essentially inferring the decay parameter. We can then discover non-random correlations when there are observed first passage lengths that vary significantly from the background distribution. With this method, we hypothesize that we can reliably extract a set of environmentally dependent correlations, and in fact of all of those correlations we deem significant are OGT dependent. Some likely relate to known thermal adaptations, such as disulfide bonding and salt-bridge formation. Yet our approach also captures unexplained, OGT-dependent effects. Hence careful exploration of these issues may yield unforeseen phenomena from extremophile genomes.

## Results

### First Passage Distributions

We analyzed first passage distributions across the entire proteomes of 76 organisms (Table S1) [12]–[13]. These organisms were chosen so that a wide range of organisms with both fully sequenced proteomes and experimentally well-characterized OGTs were included. As a result, psychrophiles, mesophiles and thermophiles are all well sampled across the known range of organism growth temperatures. We wanted to examine correlations directly dependent on OGT. First passage statistics were chosen to take into account the OGT dependence of amino acids in the background distribution, while possessing the statistical power to resolve discontiguous correlations over large lengths. These distributions are defined as the probability that the first occurrence of amino acid 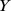 occurs 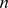 residues away from amino acid 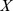 without having occurred beforehand. This is denoted by



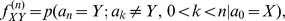
where 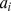 denotes the residue a distance 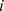 from 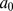 [1].

For a given chain one can calculate the typical first passage distribution in either the N-terminus or C-terminus direction, which we respectively denote by 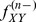 and 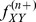. If amino acids were uncorrelated from their neighbors, this would reduce to a geometric distribution, the discrete analog of an exponential distribution [14]. In this case the probability would only depend on the frequency of 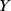 and should, therefore, reflect the OGT dependent properties of individual amino acids, allowing the OGT dependence of outliers to be clearly established. However, while many genome wide amino acid probabilities are independent, some probabilities are not [10]. Our approach addresses that possibility. As shown in the Methods Section, the log of our distribution often becomes linear after a few residues of separation, with a slope dependent on 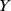 and not 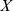. We therefore assume 
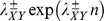
 as our theoretical background distribution for 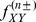, where 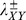 is derived from the best linear fit to the logarithm of the first passage distribution versus 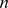. Practically, we derive it over values of 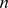 less than 50, due to noise induced by finite gene size.

**Figure 1:**
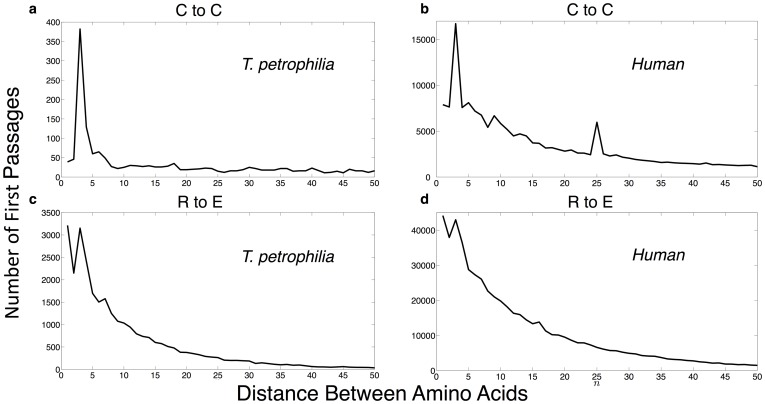
Number of first passages from cysteine to cysteine in (a) *T. petrophila* and (b) human proteomes, and from arginine to glutamic acid in (c) *T. petrophila* and (d) human proteomes.

As an example Fig. 1 shows the number of first passages as a function of 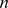 for CC and RE for two organisms, a mesophile, humans, and the thermophile *T. petrophila*. In both cases, 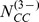 and 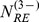 stand out from the background distribution. In the human genome, which is both longer and has longer genes, additional structures at larger 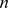 appear. However, the relative height of the peak to the background distribution is higher for the thermophile, a pattern we explore further.

Previous authors have explored different measures of pairwise correlation between residues in proteomes [15]–[16]. Liang, *et al.* calculated the frequency of chains of length 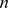 beginning with amino acid 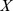 that terminate with amino acid 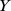 **,** divided by the frequency of 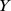 in the proteome. They recover a small subset of the correlations we find via first-passage statistics, with no measure of OGT dependence for individual correlations. However, they also declare many correlations significant which would not be considered significant by our approach, suggesting a higher false-positive rate in addition to less sensitivity. We believe this is because many of the amino acid correlations they noted, that we would deem insignificant, have individually OGT dependent frequencies. As our approach takes such effects into account explicitly by fitting the empirical background distribution rather than using a model clearly related to individual amino acid frequencies, these correlations would not be deemed significant.

Rosato, *et al.* calculated a similarly motivated odds ratio by comparing the number of times amino acid 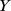 occurs 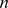 residues away from amino acid 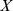 and dividing this by the expected number of times this would occur in a random proteome with the same length, number of proteins, and amino acid frequencies [17]. The only non-nearest neighbor correlation they were able to identify was that of a cysteine three residues away from another cysteine, whereas our approach reveals many other sources of such discontiguous correlation, all of which correlate to OGT.

Our method does not assume individual amino acid independence, even though that is often a reasonable assumption, and separates true discontiguous correlations from those due to many small words by excluding events where 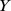 previously occurred. We believe this is a key advantage over previous approaches. It derives significance from deviations to the long-range exponentiality of these distributions, even when the geometric distribution built from individual amino acid frequencies is inadequate. As shown in the Methods Section, 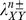 typically depends on 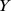 and varies with OGT when the frequency of 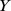 depends on OGT, and does not depend on 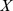. If 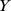 is more abundant at high OGT amino acids will typically pass to 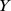 faster, and if it is less abundant more slowly. 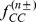 is a strong exception. Though less frequent at high OGT, cysteines average return length is faster - indicating that as cysteines become rare having them nearby at high OGT is more significant.

As a result of our improved background, all of our significant deviations from the expected exponential distribution depend on OGT. We extract these events from the logarithm of the ratio of the empirical value of 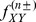 to its expected value from the underlying exponential background, termed 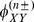. This quantity is essentially the logarithm of the amplitude of the peaks, such as those demonstrated in Figure 1, above the background distribution – representing the logarithm of a signal to noise ratio. Table 1 looks at the most commonly over-represented pairs, while Table 2 looks at the same information for under-represented pairs. Those listed have a logarithm of the amplitude height above background that is at least three standard deviations from the from the exponential background distribution within an individual organism's proteome for a minimum of 25 of the organisms studied. 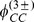 is the most significant, by a substantial margin, both in magnitude and number of organisms in which it is over-represented. Several over-represented pairs fall into categories, such as having a positively and negatively charged side chain (RE, KE, ER, EK). Leucine, the amino acid whose frequency is higher than expected from its mass, is frequently in over-represented pairs (LE, LQ, LK, LR) [10].

**Table 1:** Over-represented First Passage Statistics.

N-Terminal Direction			
Pairs	Number of Genomes	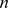	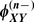
*CC*	75	3	1.545
*KE*	32	3	0.325
*RE*	41	3	0.3094
*LE*	57	2	0.2878
*EK*	26	4	0.222
*LK*	47	2	0.2207
*ER*	31	4	0.1884
*LQ*	48	2	0.1836
*LR*	35	2	0.1812
*KM*	31	2	0.1626
*IQ*	34	2	0.1583

**Table 2:** Under-represented First Passage Statistics.

N-Terminal Direction			
Pairs	Number of Genomes	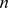	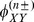
GD	29	*2*	−0.1660

We examine in detail a set of correlations along the N-Terminal direction, all of which have C-Terminal analogs. We focus on four cases from Table 1: 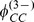, 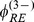 (which has similar behavior to 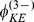), 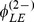 (the most commonly over-represented leucine containing pair), and 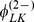, a correlation previously unobserved to our knowledge. These pairs have the highest peak values above background and have distinct behavior from each other.

### Dependence of Quantities on Habitat

**Figure 2:**
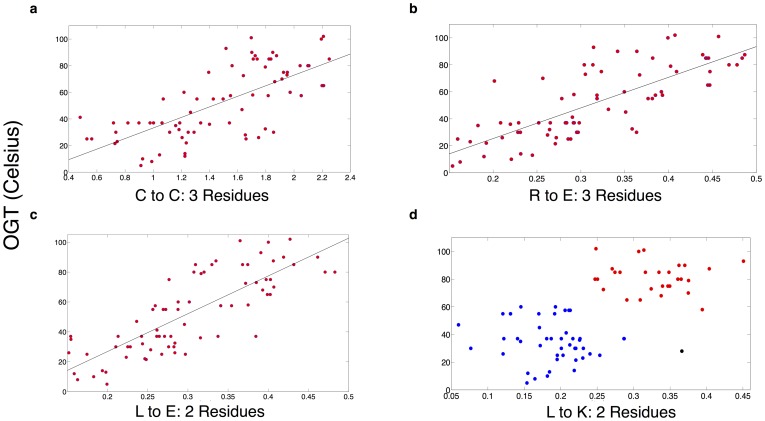
OGT as a function of the logarithm of the real to expected first passage probability, essentially the non-random parts of the amplitudes for peaks such as those in Figure 1. These are plotted for (a) CC at three residues, (b) RE at three residues, (c) LE at two residues, and (d) LK at two residues, where blue circles indicate OGT ranging from 5°C to about 60°C, and red circles represent hyperthermophilic organisms with OGT above 60°C.

To determine which signals vary with OGT, we plot, in Figure 2, the OGT for all organisms as a function of the logarithm of the peak amplitudes above background. We also calculate the linear correlation (Pearson) and Spearman correlation for these quantities, along with p-values, as described in the methods section. Figure 2 shows the dependence between OGT and 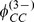 with a 0.7054 linear correlation (1.1267×10^−12^ p-value) and 0.6993 Spearman correlation (2.1146×10^−12^ p-value), with a 0.7724 linear correlation (1.1267×10^−16^ p-value) and 0.7841 Spearman (5.4453×10^−17^ p-value), and 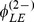 with a 0.8013 linear correlation (3.5598×10^−18^ p-value) and 0.8155 Spearman (2.9905×10^−19^ p-value). For 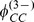, OGT dependence holds for all non-eukaryotes. Eukaryotes are in the far left cluster. Previous results did not show a measure of strength of correlation between OGT and a signal to noise parameter, which would be analogous to the quantity we define below. The figures shown in references [15]–[17] indicate that these approaches show a 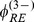 noisier dependence between the statistical quantities defined by those authors and OGT.

The significance of 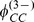 has clear interpretations. Increased clustering of cysteines may lead to an increase in disulfide bonds that can be used more frequently per residue for thermal stability or correlated effects from metal-ion binding are more prevalent [18]–[26]. As noted, cysteines become rarer at high OGT, yet seem to retain their importance for disulfide bonding [10]. For instance, many high-temperature environments tend to be more acidic. Since low pH inhibits thiol-disulfide exchange, one may expect that cysteine clustering would increase stability in the proteome of an organism in an acidic environment. Hence, there is an important tension between the rarity of cysteines at high OGT, and their potential increase in importance as a stabilizier.

Less noted, is the OGT dependence of the 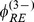. The two are oppositely charged, and may represent the stabilization of proteins via salt-bridge formation [27]–[30]. Intriguingly, we find that a great deal of information can be drawn from the unusual metabolism of organisms farthest from the best-fit line between 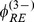 and OGT. Those furthest from that line in the upper half-plane are *S. azorense* and *I. aggregans*, and in the lower half-plane are *G. metallireducens* and *G. sulfurreducens*. This suggests a possible method by which unusual metabolic properties could be uncovered by deviation from a correlation line. Both the interpretation of why that particular amino acid correlation is proportional to OGT, and why sulfur respiration seems reflected in outliers, contain biological insight. While the former likely relates to an organism's utilization of salt-bridge formation, the later suggests a statistical measure for prediction of novel metabolism, which has practical uses such as in microbial cleaning of contaminated soil [31]–[32].


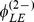
 has the strongest linear correlation with OGT. Surprisingly, the plot of 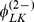 versus OGT (Fig. 2d), which has a slightly weaker, though highly significant, temperature dependence (0.6278 linear with 1.2765×10^−9^ p-value, 0.6042 Spearman with 7.5086×10^−9^ p-value), clusters these organisms into two main groups. One set, in red, contains most thermophiles while the other, colored blue, contains lower OGT organisms. This suggests a genome wide statistical property can classify thermophiles from non-thermophiles. Moreover, the quantity 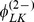 lacks an obvious interpretation.

## Disscusion

We have shown that first passage distributions identified multiple OGT dependent statistical measures, indicating that they are a natural language for the inference of environmentally dependent amino acid correlations. We found a general exponential decay of first passage distributions. As indicated in Table S2, the inferred exponential first passage distribution often is, though not always, what one would expect if an amino acid is uncoupled from its neighbors.

We believe this is analogous to the rapid mixing that can generate exponential first passage distributions in a Markov Chain [33]–[35]. When an amino acid is not independent, it could appear in small, weak local groupings, enhancing the probability of staying near a small set of states before undergoing a transition to a forbidden state, making the decay time longer when independence is violated [33]. Moreover, in microbes, noise due to small genome size may mask richer behavior such as short-range deviations from exponentiality, resulting in stretched exponential distributions. Future studies should also compare the OGT dependent discontiguous correlations found by this measure to other approaches to inferring long-range correlations from primary amino acid sequences [36]–[37].

First passage distributions identified multiple OGT dependent statistical measures, many of which were unknown or previously less well characterized. Deviants from the general pattern of exponential decay indicate significant correlations that appear to be related to physical interactions such as disulfide bonding, from an enhancement of cysteines three amino acids away from cysteine, and salt-bridge formation, from sulfur metabolism. Moreover they identified a set of OGT dependent measures lacking a clear physical explanation, implying that this approach may be used to identify temperature sensitive amino acid correlations to uncover important properties of proteins in extreme environments.

Other than the cysteine peak, likely related to disulfide bonding, the first passage statistics singled out in Table 2a may also indicate specific enhancements of alpha helices that appear on the surface of a protein [38]. The significant values at a residue distance of two, one of which was the stringest observed correlation, all contain one hydrophobic and one hydrophilic residue, likely indicating one residue facing the inside and one facing the outside of a protein alpha helix. For those at length three and four, other than the cysteine peak at three residues, all residues are polar (REK), indicating alpha-helices facing the outside of a protein. In both cases, there would be an interaction with the external environment generating the observed OGT dependence, along with predictions about which specific residue patterns are enhanced. Further sequencing and characterization of extremophile habitats, may uncover new, unexpected effects, while furthering our understanding of what is a typical protein in a given environment.

## Methods

### Correlations

In general correlation coefficients were calculated using the “corr” functions in Matlab for the Pearson and Spearman correlation coefficients. P-values were calculated using Student's t-test for the Pearson correlation and a permutation test for the Spearman correlation, also using the Matlab “corr” function. Typically both coefficients are quite close in value. When no other method is indicated in the text an “*r*” value indicates the Pearson correlation.

### Empirical Evidence for Exponential Distribution

To test the hypothesis that the appropriate background distribution for the amino-acid first passage distributions defined in the text is indeed exponential, we performed the following set of tests. Each test was performed independently for each organism. Organisms were never compared under the same statistical test for these p-value calculations.

For each first passage distribution, 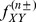, we calculated the linear correlation coefficient between the value of 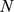 and the logarithm of the first passage probability. Again, this was done separately for each organism. A p-value was assigned to each correlation to determine the significance of that linear correlation using Student's t-distribution for a transformation for the correlation. Both tasks performed using the function “corrcoef” in Matlab, using Pearson correlation.

In Table S2, for 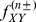, the above mentioned p-values are explored for each of the 400 amino acid pairs that were tested separately for each organism. Even if multiple hypothesis testing is strictly taken into account, by treating each of the 76 cases of 400 hypothesis separately for a p-value cutoff of 0.05/30400 for a -log(p-value) of 13.318, there are only a 11 cases where the linear correlation is insignificant. All of them involve low frequency amino acids, and therefore may be due to large fluctuations. The minimum, maximum and medium values of -log(p-value) are shown in Table S2 for the 400 possible values of 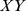. Of the significant values, eight of them are between tryptophan and cysteine, two are between cysteine and tryptophan, and one is between methionine and cysteine. All occur in extremophiles where cysteines are rare and have large fluctuations.

Also shown in this table are the estimates for the average first passage, 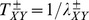 as derived from the background distribution, the standard deviation, and minimum and maximum values. Specifically we show the case 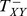. In most cases the values are at least tens of residues (than the typical length at which amino acid word statistics are significant [10]), and depend on 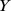 only. To establish the hypothesis that dependence is typically on 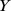 rather than 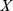, we compared the median value of 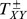 to the values of 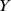 using the Kruskal-Wallis test, and also compare them to the values of 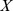. When compared to 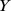 there was a very significant p-value of less than 10^−71^. When compared to 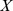 there was no significance. This would imply that an analogous process the “mixing” mechanism for exponentiality in Markov processes is responsible for this effect [33]–[35]. In this case the distribution becomes stationary well before all states are explored.

If all amino acids were independently distributed according to their empirical frequencies, the first passage distribution would be a geometric distribution determined by the frequency of amino acid 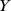. In that case the 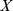 independent decay constant, 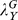, would be
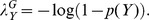



Associated with this is a geometric decay length, 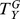. If 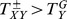, the observed exponential distribution decays more slowly than in the case when amino acids are independently distributed and, likewise, it decays would decay faster if 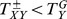. In Table S2, we show the median value of the ratio 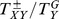. In many cases the ratio is quite close to 1, indicating that the amino acids are basically independent of each other. However for those quantities to be calculated correctly, occurrences due to significant peaks would have to be removed, and a new background frequency inserted into the above formula.

### Temperature Dependence of Exponential Decay

The Spearman correlation between the average expected length of first passage 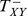 and OGT is shown in the final column of Table S2. All distributions where 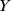 is equal to V, E or Y have a negative correlation less than −0.5. These amino acids are more abundant at high OGT, and therefore an amino acid will typically arrive at them more quickly when this occurs, causing the mean length of first passage to decrease. Likewise, H, T and Q, which become rare at higher OGT, all have longer means. The strongest exception to this rule is 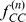. Despite the fact that cysteines typically become rare at higher OGT, its mean length of first passage to another cysteine becomes shorter. The implication is that at high OGT, while C becomes less frequent it is more and more important that cysteines appear near each other. In addition to the significant spike at 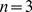, cysteines likely have many weak correlations with each other at higher OGT at different length scales. Such an effect only reinforces taking into account the background distribution in a manner that does not assume complete independence of residues.

## Supporting Information

Table S1 **Organisms studied, along with optimal growth temperature (OGT).**
(XLSX)

Table S2 **Range of first-passage statistics for four hundred amino acid pairs.** Maximum, minimum and median values are listed for within organism p-values and typical first-passage lengths across the organisms studied. The median values for the ratio of the observed first-passage length to what one would expect from a geometric distribution, and the Spearman correlation are also listed.
(XLSX)
