# Correction: Using First Passage Statistics to Extract Environmentally Dependent Amino Acid Correlations

**DOI:** 10.1371/journal.pone.0113943

**Published:** 2014-11-12

**Authors:** The PLOS ONE Staff

There are errors in the author affiliations. The affiliations should appear as shown here:

Benjamin D. Greenbaum^1,2^, Pradeep Kumar^4^, Albert Libchaber^2,3^

1 Departments of Medicine, Division of Hematology and Medical Oncology, and Pathology, and the Tisch Cancer Institute, Icahn School of Medicine at Mount Sinai, New York, New York, United States of America, 2 The Simons Center for Systems Biology, Institute for Advanced Study, Princeton, New Jersey, United States of America, 3 Center for Studies in Physics and Biology, The Rockefeller University, New York, New York, United States of America, 4 Department of Physics, University of Arkansas, Fayetteville, Arkansas, United States of America
